# Informative graphing of continuous safety variables relative to normal reference limits

**DOI:** 10.1186/s12874-018-0504-z

**Published:** 2018-05-16

**Authors:** Christopher D. Breder

**Affiliations:** 1 Division of Neurology Products, Food and Drug Administration, Silver Spring, MD USA; 2 Advanced Academic Programs in Regulatory Science, Krieger School of Arts and Sciences, Johns Hopkins University, Rockville, MD USA; 3 Center for Drug Safety and Effectiveness, Bloomberg School of Public Health, Johns Hopkins University, Rockville, MD USA

**Keywords:** Scaling, Multiples, Multiplicative inverse, Reference range

## Abstract

**Background:**

Interpreting graphs of continuous safety variables can be complicated because differences in age, gender, and testing site methodologies data may give rise to multiple reference limits. Furthermore, data below the lower limit of normal are compressed relative to those points above the upper limit of normal. The objective of this study is to develop a graphing technique that addresses these issues and is visually intuitive.

**Methods:**

A mock dataset with multiple reference ranges is initially used to develop the graphing technique. Formulas are developed for conditions where data are above the upper limit of normal, normal, below the lower limit of normal, and below the lower limit of normal when the data value equals zero. After the formulae are developed, an anonymized dataset from an actual set of trials for an approved drug is evaluated comparing the technique developed in this study to standard graphical methods.

**Results:**

Formulas are derived for the novel graphing method based on multiples of the normal limits. The formula for values scaled between the upper and lower limits of normal is a novel application of a readily available scaling formula. The formula for the lower limit of normal is novel and addresses the issue of this value potentially being indeterminate when the result to be scaled as a multiple is zero.

**Conclusions:**

The formulae and graphing method described in this study provides a visually intuitive method to graph continuous safety data including laboratory values, vital sign data.

## Background

Graphic presentations have long been recognized as a useful method to present clinical trial data [1, 2]. This is particularly true for safety data because clinical studies are not typically designed to inferentially test safety and tolerability data. The International Conference on Harmonization (ICH) guidance to industry on Statistical Principles for Clinical Trials recommends graphic inspection for safety and efficacy, data, including dose-response relationships [3].

Accurately interpreting the graphs of safety data may be challenging because of differences in laboratory reference ranges between testing sites, genders, and age groups. For example, the reference range for plasma alkaline phosphatase concentrations varies between age, gender, and testing center. The reference ranges of many pediatric laboratories and vital sign tests vary considerably, particularly before adolescence, so interpretation of the graphical display from a clinical trial of one or more year’s treatment duration in the very young can be particularly challenging. Graphing the actual values can also result in a misleading mixture of normal and abnormal results in the regions of the upper and lower limits of normal because of overlapping differences in these ranges. These differences are sometimes due to variances in assay techniques or demographics of the reference population between sites.

Several studies recognize these issues and suggest using multiples of the reference range limits instead of the actual values, although these descriptions are typically limited to demonstrating values relative to the upper limit of normal (ULN). When this approach is applied to data with abnormal values above and below normal range, the normal and abnormal low values are ‘compressed’ between 1 and 0 multiples of the ULN, while the abnormal high values have, at least theoretically, no upper limit [4]. This is not usually concerning when working with data from biomarkers of cell injury, such as lactate dehydrogenase (LDH), which can have multiples in the thousands over the ULN and where a low value is not clinically concerning. However, other analytes, such as serum glucose or hematocrit, often have abnormal values higher than the ULN and below the LLN. One approach to the problem of displaying values above and below the normal range is to plot the standard deviations of the sample mean; however, one is often testing a sample where almost all of the subjects have abnormal values, and so the result would look almost the same as if a similarly homogeneous population of normal subjects was tested.

The objective of this study is to develop a method of graphing continuous safety data in a manner so as to address the issue of compression, particularly with datasets having large numbers of data below the LLN and for data with abnormal values both above the ULN and below the LLN. The problems encountered with graphing the actual values using standard techniques are first demonstrated and then formulas are derived to graph data in a manner that addresses scalability above and below the normal range and to produce graphs that are visually intuitive.

## Methods

### Datasets used for methods development and analyses

Methods for graphing were developed using a mock labs data set (MDLD.JMP) generated in the software program JMP® (v.13, SAS Institute). Lab values were produced using a formula-based random numbers generator between numeric ranges seen with actual testing of an analyte that may have abnormal high and low values, such as transferrin. Additional values of 0 (zero) were substituted for demonstration purposes. Demographic column variables such as unique subject identifier (USUBJID), Testing Site (SITE), Planned Treatment (TRTP), and Visit sequence (VISIT) are also included in the dataset. The mock dataset is constructed so that subjects assigned to be tested randomly at each of the four sites during the course of the trial. Columns are created for the ULN and lower LLN limit of normal reference ranges for each value (RESULT) based on the testing site at the time of the laboratory blood draw based on those publically available for transferrin from the 4 testing sites (SITE) (Table 1). An abnormal flag column (ABNFL) is added to the dataset to designate the RESULT as being normal (N), abnormally high (H), or abnormally low (L).

**Table 1:** Reference Limits for Plasma Transferrin used in the Analysis of the Mock Laboratory Dataset

Site # / Name	Source	Upper Limit of Normal (mg/dL)	Lower Limit of Normal (mg/dL)
1 / Quest Diagnostic Center, Baltimore site (QBA)	http://www.questdiagnostics.com/testcenter/BUOrderInfo.action?tc=891X&labCode=QBA	341	188
2 / University of Kentucky Health Care	http://app.mc.uky.edu/pathology/refsearch/details.asp?id=541	360	200
3 / University of Utah Health Sciences Library	http://library.med.utah.edu/WebPath/EXAM/LABREF.html	360	212
4 / University of California, San Francisco Clinical Laboratories	http://labmed.ucsf.edu/labmanual/db/data/tests/576.html	360	182

*Abbreviations*: *mg/dL* milligrams per deciliter

### Graphical display of untransformed lab datasets

Graphs are first created for the untransformed data using the variable for time (e.g., VISIT in MDLD.JMP) as a continuous variable for the X-axis. The RESULT is a continuous dependent variable on the Y-axis. For the purposes of demonstration, lines for the normal reference limits are added to the Y-axis designating the ULN and LLN for each of the laboratory testing sites. Colored row states are created designating H as blue + signs, N as orange dots, and L as black inverted triangles.

## Results

### Graphic inspection of untransformed data

**Fig. 1:**
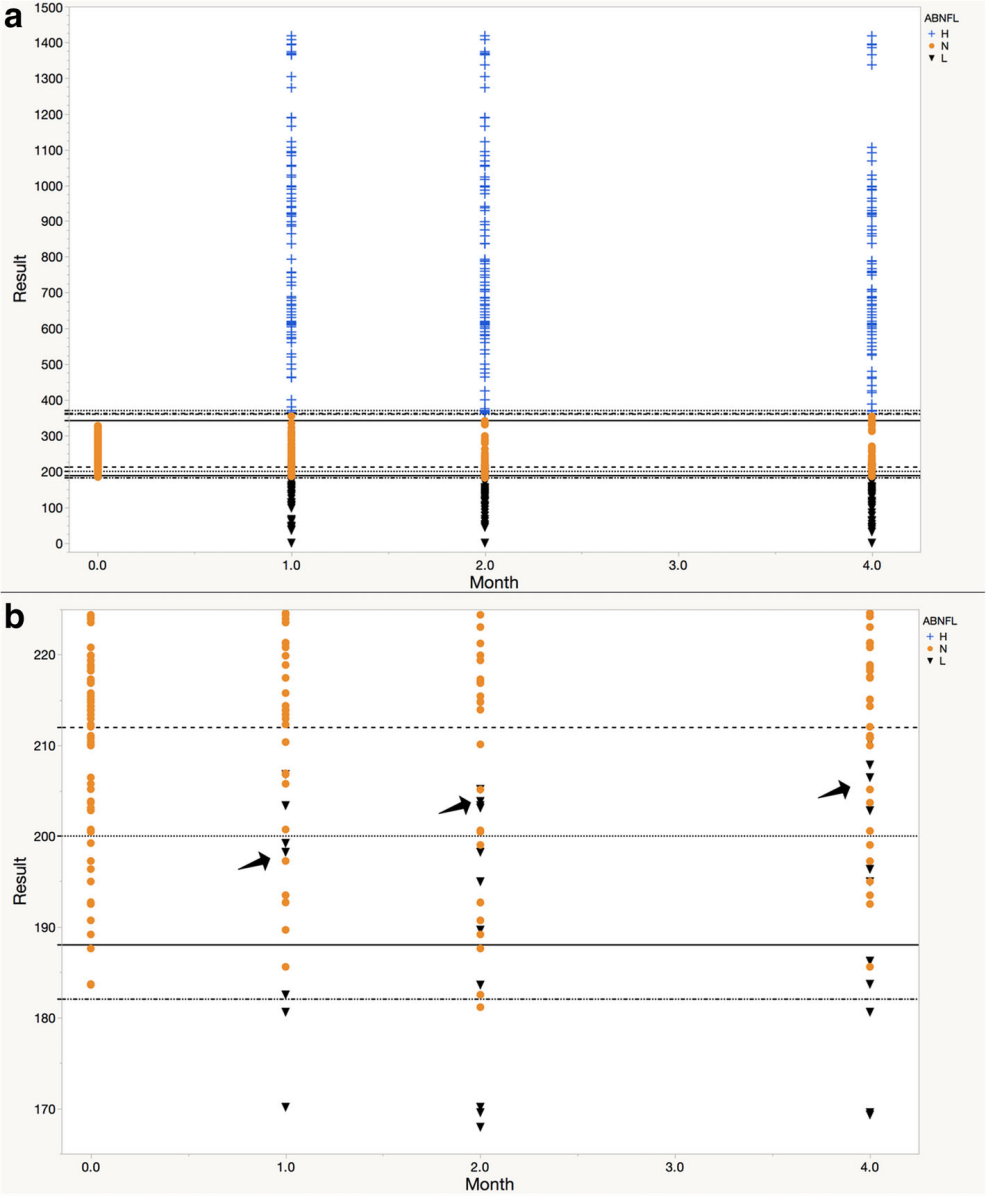
Graphical View of the Untransformed RESULT Values in the MDLD dataset. **a** Graphical View of RESULT Values Versus Time. **b** Spatial Blending at the Level of the Lower Limits of Normal of the Four Testing Sites. Mock data from the MDLD dataset (RESULT variable) are plotted on the Y-axis. Time (VISIT variable) is assigned to the X-axis. Values above the ULN are marked by a blue “**+**”, RESULT values below the LLN are marked by a black “▼”, and values between and inclusive of the LLN and ULN are designated by an orange “”. Reference ranges for the four sites are provided as (^___^) Site 1, (^…..^) Site 2, (^_ _ _^) Site 3, and (^.._.._^) Site 4. Note the orange dots signifying normal values are intermixed with the black inverted triangles that represent values lower than normal (see black arrows). Abbreviations – ABNFL, abnormal flag; H, abnormally high RESULT, L, abnormally low result; mg/dL, milligrams per deciliter; N, normal RESULT

Figure 1 is a presentation of RESULTS from the MDLD dataset by visit month with site-specific reference lines for the ULN and LLN. At the lower magnification, one can appreciate that values below the LLN are relatively compressed compared to those above the ULN (Fig. 1a). One can appreciate at higher magnification that the region of the LLN reference limits contains a mixture of normal and abnormally low values (Fig. 1b); this may be seen at the level of the values around the ULN lines as well. These issues may be exacerbated in trials with multiple sites (e.g., if sites use different assays), with pediatric patients, and where both genders are represented (e.g., if there are gender-specific reference ages).

### Derivation of formulas to transform data for graphing

The formulae needed to graph the possible scenarios (L, N, H) are found in the following set of equations:

Equation 1 Multiples of ULN (**xULN** in the MDLD dataset) for graphing “H” values – This is the standard formula for ULN

$$ xULN\kern0.5em =\frac{RESULT}{ULN} $$

Equation 2 – Normal results (**NLRES**), for graphing values equal to or falling between the ULN and LLN. This is a standard formula for scaling or normalizing, results between 2 numbers [5, 6], that has been adapted for this case where the boundaries are the ULN and LLN

$$ NLRES\kern0.5em =\kern0.5em \left(\left(\frac{RESULT- LLN}{ULN- LLN}\right)\kern0.5em \times \kern0.5em 2\right)\kern0.5em -\kern0.5em 1 $$

Equation 3 - Multiples of LLN (**xLLN**) for graphing “L” values – This is the ‘multiplicative inverse’ of the ULN-type formula, so the results graphically fall below − 1, where the LLN is situated

$$ NLRES\kern0.5em =\kern0.5em \left(\frac{LLN}{RESULT}\right)\kern0.5em \times \kern0.5em -\kern0.5em 1 $$

Equation 4 - Multiples of LLN (MI) for graphing “L” values where the RESULT is equal to 0 – A special case of the xLLN formula is needed because the RESULT is in the denominator of the MI formula and in the case where the result is 0, the MI would be undefined, or ∞. The Multiplicative Inverse to be used when the result equals 0 is designated as ***MI***_***0***_; the final formula is presented below and its derivation follows

$$ {MI}_0\kern0.5em =\kern0.5em {MI}_{max}\kern0.5em \times \kern0.5em \left(\frac{LLN}{LLN-{RESULT}_{max}}\right)\kern0.5em \times \kern0.5em - 1 $$

MI_0_ will be some distance MI_A→0_ beyond MI_max_, the multiplicative inverse of the abnormal value, A, with the greatest distance from its LLN (see Eq. 3). The variables in this last statement may be represented as

4.1a $$ {MI}_{max}\kern0.5em =\kern0.5em \frac{B}{A}\kern0.5em =\kern0.5em \left(\frac{LLN}{RESULT_{max}}\right)\kern0.5em \times \kern0.5em -\kern0.5em 1 $$

4.1b $$ {MI}_{A\to 0}\kern0.5em =\kern0.5em \frac{B}{B-A}\kern0.5em =\kern0.5em \left(\frac{LLN}{LLN-{RESULT}_{max}}\right) $$

When these are added together, they equal the multiplicative inverse of 0.

4.2 $$ {MI}_0\kern0.5em =\kern0.5em \left(\frac{B}{A}\kern0.5em +\kern0.5em \frac{B}{B\kern0.5em -\kern0.5em A}\right)\kern0.5em X\kern0.5em - 1 $$

Equation 4.2 simplifies algebraically to the following:

4.3 $$ {MI}_0=\left(\frac{B^2}{A\left(B\kern0.5em -\kern0.5em A\right)}\right)\kern0.5em X\kern0.5em - 1\kern1em \Rightarrow \kern1em \left(\left(\frac{B}{A}\right)\times \left(\frac{B}{B-A}\right)\right)\times - 1 $$

Substituting back the terms for A and B, the final formula is demonstrated.

4.4 $$ {MI}_0\kern0.5em =\kern0.5em {MI}_{max}\kern0.5em \times \kern0.5em \left(\frac{LLN}{LLN-{RESULT}_{max}}\right)\kern0.5em \times \kern0.5em - 1 $$

### Formulae needed to graph transformed lab datasets in JMP

Graphs are created for the transformed data using the methodology described for the untransformed data except that the continuous dependent variable on the Y-axis is a conditional formula (e.g., If, Then…) containing the formulae for the following:

- Multiple of the ULN for RESULTS that are abnormally high,
- Normal RESULTS scaled between 1 and − 1,
- Multiple or multiplicative inverse of the LLN for RESULTS that are abnormally low but greater than 0, and for the
- Multiple or multiplicative inverse of the LLN for RESULTS that are abnormally low but equal to 0. Reference lines are added to the Y-axis at (0, 1) and (0, − 1) to designate 1× multiple of the ULN and LLN, respectively.

**Fig. 2:**
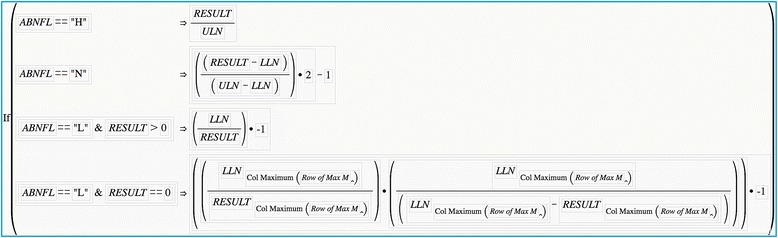
Formulae to Graph the Transformed RESULTS Y-Axis Data as they Appear in the JMP Formula Editor Box. Abbreviations – ABNFL, abnormal flag column; COL, column; H, abnormally high RESULT, L, abnormally low result; LLN, lower limit of normal; Max M, maximal value of the multiplicative inverse of the LLN; N, normal RESULT; ULN, upper limit of normal

Figure 2 presents these formulae as they appear in the JMP Formula Editor. The steps necessary to arrive at the formula for the MI_0_ in the JMP dataset include:

1. Create columns for the **RESULT** and corresponding **LLN**
2. Create a Column **MI** using the formula LLN ÷ RESULT
3. Create a Column **Max M** using the formula Col Maximum(**M**). This is done using Col Maximum from the Statistical function of the Functions (grouped) dialogue box and designating Column M as the variable of operation.
4. A column (**Row of Max M**) with the conditional formula, IF [**M**] = [**MAX M**] → [ROW()] specifies the row containing the maximum M for the next step. All of the cells in this column will appear to be missing values, except the rows corresponding to the maximum M value.
5. The formula for MI_0_ is written as one of the scenarios (last line, Fig. 2) for the conditional function that will be graphed on the Y Axis. The MIo formula makes use of Subscript from the Row function of the Functions (grouped) dialogue box to exclusively use the RESULT and LLN of the maximum multiple of the LLN.

### Graphical display of transformed data

**Fig. 3:**
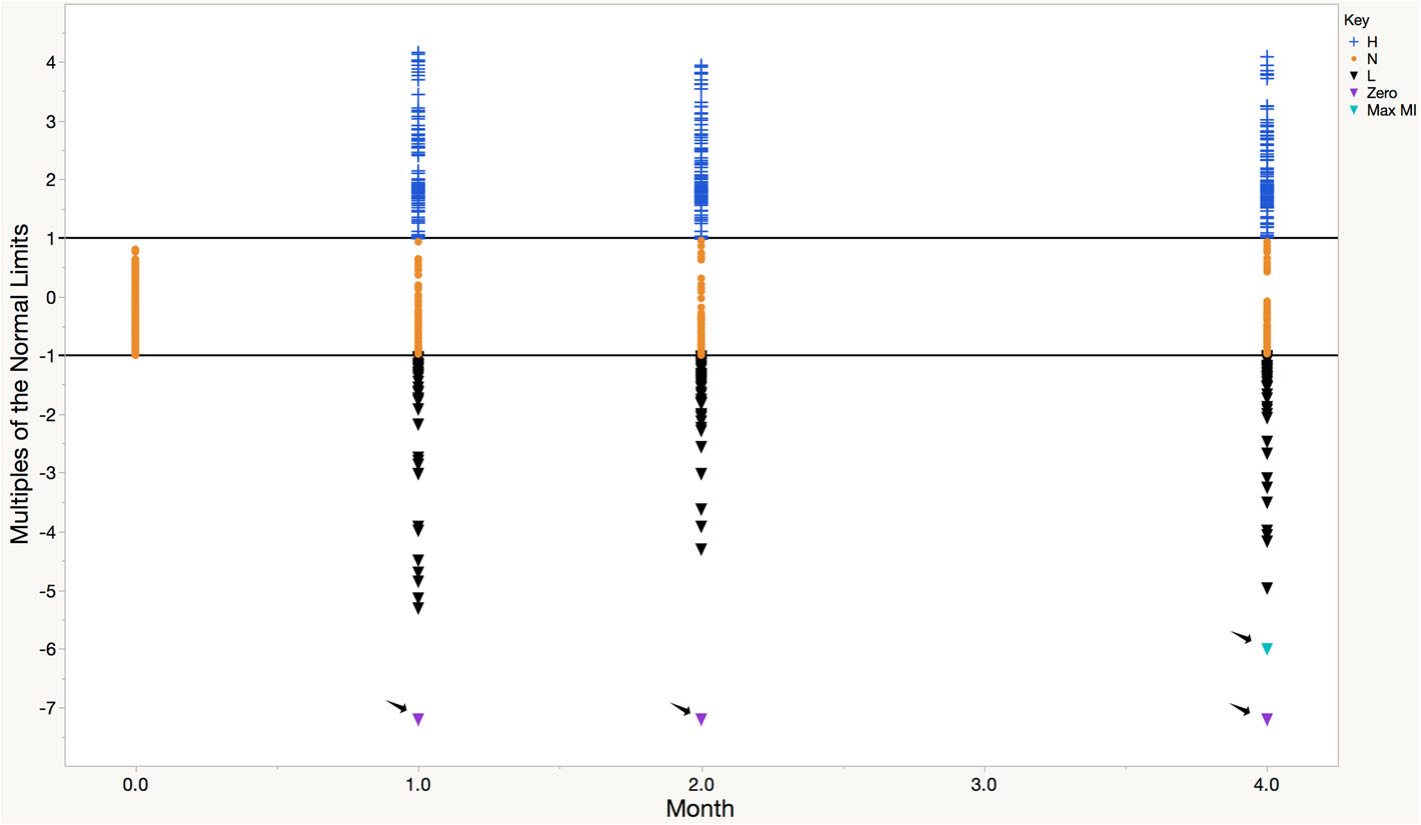
Transformed RESULT values graphed versus Time. Values above the ULN are marked by a blue “**+**”, RESULT values below the LLN are marked by a black “▼”, and values between and inclusive of the LLN and ULN are designated by an orange “”. Reference lines (^___^) are provided at Y-Axis at (0, 1) for the ULN and (0, − 1) for the LLN. Black arrows points to the points corresponding to inverted purple triangles (“”) where the multiple of the LLN for RESULTS of zero were imputed and to inverted light blue triangles representing the value of MI_max_, the abnormal low value furthest from the LLN (“”). Abbreviations – H, abnormally high RESULT, L, abnormally low result; Max MI, maximal value of the multiplicative inverse of the LLN; N, normal RESULT

Figure 3 displays the results when graphing continuous variables where values that are both abnormally low and high are presented with a similar scale. Normal values are displayed in a linear distribution between the ULN and LLN. The black arrows pointing to purple inverted triangles indicate the locations where the multiplicative inverse of the LLN is located for RESULTS of zero based on the equation in Eq. 4.3. The black arrow pointing to the light blue inverted triangle indicates the location of the multiplicative inverse, MI_max_, described in Eq. 4.1a. This is the point that is used to derive the position of the MI_0_, as demonstrated in Eq. 4.

## Discussion

### Advantages of graphic presentation of safety data

Graphic presentation of safety variables provides a means to enhance the accuracy of signal detection. Evidence for this has been reviewed in a recent publication by Michard [7]. Businesses, such as aeronautics, have long known this, designing key displays with graphics rather than numeric displays. The advantage of graphical presentation has been systematically studied with respect to vital sign data in the disciplines of cardiology [8, 9], anesthesiology [10], and critical care medicine [11]. Evaluation of clinical data in graphic form or a combination of numeric and graphic data has been shown to produce improved data interpretation speed and accuracy in several clinical scenarios. In one study of home blood pressure monitoring, this difference in monitoring resulted in differences in patient management, as measured by medications used. While definitive evidence was not found that graphic display could have a meaningful effect on mortality, several studies mentioned above have demonstrated significant improvements in the detection of myocardial ischemia or acute coronary syndrome, which can be fatal.

### An unmet need in graphical presentation of data

Potential discrepancies and deficiencies in standard numeric reporting of clinical safety data values have been long recognized. In the 1970’s, Duboff reported creatine kinase values for members of a family with malignant hyperthermia that differed based on the testing site [12]. In this same period of time, the College of American Pathologists’ Enzyme Survey demonstrated that results of liver enzyme tests varied by site, presumably because of differences in instruments, reagents, and assay conditions [13, 14]. The findings from these studies in the 1970’s coincided with a revolution in the use of graphical presentation of data, prompted largely by the work of Cleveland [1, 15, 16] and similar biometricians.

Recent attention to the use of graphics in the presentation of safety data has brought refinements [17, 18] including the use of individual patient graphic profiles [19]; however, limitations and misperceptions still exist.

Rodbard discussed potential issues in perception when viewing data graphed for analytes such as glucose, which may have abnormal data that are both above the ULN and below the LLN. Despite being potentially more critical in the acute setting, hypoglycemic values may be overlooked when presented graphically because, in contrast, hyperglycemic values can be so relatively large that the appearance of the low values does not appear meaningfully different than those that are normal when graphed using standard methods [20]. While the concept of *compression* discussed by Bottger and Balzer [4] relates more to distortions caused by two unequal stretching operations, the phenomenon described by Rodbard et al., seems to describe compression of abnormally low values relative to the scaling of those above the ULN.

### Previous proposals for graphing safety data

Several authors have proposed alternative methodologies for graphing. The most commonly used is based on multiples of the ULN. This method has the most utility for monitoring plasma levels for enzyme biomarkers of tissue damage such as AST, ALT, and CPK. One of the original methods proposed “centrinormalized units” that divide the ULN by 100 and scale all results by this factor so the normal range would always be 100 (upper limit) to LLN*100/ULN (lower limit) [21]. More recently, a system based on the ULN has been proposed as the method to evaluate drug-induced liver toxicity based on ‘Hy’s Law’ [22]. A closely related alternative methodology proposes to use both multiples of the ULN and baseline in a statistical outlier technique to define the critical boundaries of toxicity methods [23]. While the ULN-based methods have value in certain analyses, this methodology would not be useful when plasma analytes or vital sign measurements have values below the lower limit of normal. Normal values would also be scaled based on the ULN, which would make these data difficult to interpret.

Methods of graphing based on the standard deviation (SD) of data have also been proposed, where for example, data falling outside of 2 [24] or 3 [25] standard deviations of the mean would be considered abnormal. This method has the advantage of being able to described data above the ULN and LLN in a scale that is equivalent; however, the utility of this method is limited because not all sample populations are equivalent between hospital or lab testing sites and the population that comprises the mean ± 2 SD is not necessarily normal.

Rodbard [20] proposed use of a semilogarithmic plot to triple the percentage of the vertical axis allocated to values below the LLN, as is encountered in cases of hypoglycemia, while at the same time compressing the region of the graph containing hyperglycemic values. This method has value in expanding the detail for values below the LLN, yet it is not optimal for simultaneously visualizing data containing low, normal and high values.

### Scaling of values with the proposed method

The primary objective in developing this method was to provide a means to graph data where values below the LLN were scaled the same as those above the ULN. The first step was relatively simple; transforming abnormally high values above the ULN is typically done by dividing the value by the ULN reference limit. The scaling of results below the LLN is not typically found in literature and maintaining a scalar, visually intuitive display is not as simple as the ULN. The multiple of LLN is multiplied by − 1 in the last step before graphing so that it occupies a position equidistant to normal values as those above the ULN; this final value is termed the multiplicative inverse. While the issue of zero in the denominator would not exist if the formula was configured the same as that of the ULN (e.g., RESULT/LLN), this relationship cannot be used because zero values would result in a multiple equal to zero, which would place the point in the middle of the normal values, which are scaled between 1 and − 1.

The calculation of the multiples of the abnormal low value below the LLN will be problematic when the RESULT equals zero, since this would make the multiplicative inverse an undefined number using the equation LLN/RESULT. This situation is not typically found with certain data, such as vital signs or chemistry laboratories, where having a result of zero is not typically observed. However, there are laboratories where zero is encountered (e.g., the absolute neutrophil count in profound neutropenia) and so the issue must be addressed. A RESULT of zero would intuitively have a multiple greater than any non-zero value in the column. An additional quantity would need to be added to the column maximum of the greatest multiple of the LLN equal to a ratio that is based on the LLN and the value remaining after the smallest result is subtracted from the LLN, as is demonstrated in the derivation of Eq. 4.

### Limitations and concerns

Transformation of data almost always has potential issues with the audiences’ ability to comprehend the relationship of numbers when graphically displayed. Log transformation is one of the procedures most often used and it results in a display that may be uninterpretable by those outside of the scientific field. The method presented in this study has the advantage that it is visually intuitive; abnormal data of equal magnitude are equidistant from the normal reference limits. Most who routinely work with data are so accustomed to compression of results less than the lower limit of normal displayed in the Cartesian system relative to that above the upper limit of normal that visualization of the data in a more naturally expanded state will require familiarization. The advantage of this technique, particularly, the ability to look at data with multiple reference ranges in the same graph, will make such effort worthwhile. The method to graph points with a value of zero is not likely to be used often. Other techniques such as inclusion of a ‘zero-corrector’ (such as the use of ½ when an incidence is zero in the calculation of relative risk) may be simpler though this introduces error in the visual display.

## Conclusions

A method to graph continuous safety variables is presented. This method addresses the issues that arise when graphing values that have different reference limits, such as sometimes seen with differences in gender, age, and testing site. The graphing method presents novel approaches to the plotting of normal values in graphs of multiples of the reference limits based on a classic scaling equation. The method also includes a novel approach to scaling the multiples of the abnormal low value below the LLN when the result is zero.

## Abbreviations

A: Greatest non-zero number below LLN used in the derivation formula
ABNFL: Abnormal flag
ALT: Alanine transaminase
AST: Aspartate transaminase
B: LLN used in the derivation formula
COL: Column
CPK: Creatine phosphokinase
H: Abnormally high value
ICH: International Conference for Harmonization
L: Abnormally low value
LDH: Lactate dehydrogenase
LLN: Lower limit of normal
LLN_max_: Lower limit of normal for the RESULT_max_
M: MI used in the JMP column formula to derive MI_0_
Max M: MI of the RESULT_max_
MDLD.JMP: Mock labs data set
mg/dl: Milligrams per deciliter
MI: Multiplicative inverse
MI_0_: Derived multiplicative inverse of zero
MI_A → 0_: Distance from MI_max_ to MI_0_
MI_max_: Multiplicative inverse of the non-zero abnormal value, A, with the greatest distance from its LLN
N: Normal result
NLRES: Values equal to or falling between the ULN and LLN
RESULT_max_: Non-zero number greatest magnitude below LLN, also designated as A
SD: Standard deviation
TRTP: Planned treatment
ULN: Upper limit of normal
USUBJID: Unique subject identification
xLLN: Multiples of LLN
xULN: Multiples of ULN
